# Five months into conflict: near total collapse of cancer services in Sudan

**DOI:** 10.3332/ecancer.2023.128

**Published:** 2023-10-09

**Authors:** Salma S Alrawa, Esraa SA Alfadul, Moawia Mohammed Ali Elhassan, Nazik Hammad

**Affiliations:** 1 Faculty of Medicine, University of Khartoum, Khartoum, Sudan; 2 Station Of Medical Essentials (SOME) Institution, Khartoum, 11111, Sudan; 3 Department of Oncology, National Cancer Institute, University of Gezira, Sudan; 4 St Michael’s Hospital, University of Toronto, Toronto, ON M5B 1W8, Canada

**Keywords:** cancer care, conflict, war, developing countries, sudan

## Abstract

The onset of the Sudanese military conflict on April 15, 2023, has had a profound impact on the healthcare infrastructure across the entire nation. While wars impact all individuals with non-communicable diseases, cancer patients are particularly vulnerable. The war has influenced the treatment of Sudanese cancer patients in many ways. It has disrupted and delayed the diagnostic process, suspended surgery, and all forms of cancer management such as radiotherapy, chemotherapy and palliative care. This unfortunate situation has likely worsened the outcomes for many Sudanese cancer patients. This editorial reflects the situation of cancer services in Sudan post conflict.

The military conflict that erupted on the 15th of April 2023 in Sudan continues to claim lives and has caused the displacement of 5.1 million Sudanese people. Additionally, it has resulted in severe food insecurity and limited access to essential services, including health care, mainly in the states affected by the conflict [1]. The entire health system is on the verge of collapse, with more than 70% of hospitals in conflict areas currently out of service [2]. Consequently, oncology services have been significantly affected, resulting in disastrous outcomes.

Sudan has been a pioneer in cancer care delivery in Sub-Saharan Africa, establishing an oncology center as early as 1964 [3]. For several decades, government-owned oncology centers have been providing chemotherapy and radiation therapy (RT) to cancer patients as part of universal health coverage. Sudan has a total of fifteen public and private oncology centers. However, only four of these centers offer RT services. Unfortunately, the present conflict has severely disrupted these services, particularly in the Khartoum state and the western region, which is the epicenter of the ongoing military conflict. As a result the country now operates with only six centers; among them, only two offer comprehensive oncology care including chemotherapy and RT [4]. These two institutions are the National Cancer Institute (NCI) in Wad Madani in the Gezira State, owned by the government, and the privately owned Merowe Oncology Center in the Northern State.

In the conflict zones, the ongoing war in Sudan has severe consequences not only on people’s daily lives but also on their healthcare systems. Health infrastructure has been affected with reports of entire hospitals being destroyed, resulting in limited access to medical facilities. Among the many challenges faced, providing adequate cancer care is particularly difficult. Other inconceivable challenges that cancer patients face are loss of family structure and support with an uncertain future, war-related stress, shortages in electricity and water supply, lack of transportation, and financial devastation. Therefore, cancer patients currently undergoing oncologic treatment have travelled to neighbouring states, such as Gezira state and River Nile state, to ensure the continuation of their treatments.

Between April 15, 2023, and August 31, 2023, with the total cessation of oncology services in Khartoum, there has been a significant rise in the number of new cancer patients seeking treatment at oncology centers in nearby states compared to the same period last year. At the National Cancer Institute (NCI), the average number of new cancer cases registered has almost doubled from 1560 cases in the previous year to 2980 cases. Similarly, at the Tumour Therapy and Cancer Research Center in Shendi, there has been an increase from 163 cases to 791 cases. The East Oncology Center in Elgadarif treated 165 cases last year, which has risen to 337 cases from the start of this military conflict. Additionally, the average number of new cancer cases registered at the Eldaman Oncology and Radiotherapy Center in Merawi has risen from 217 cases to 397 cases. This surge in patient load occurred alongside an acute shortage of medical supplies, putting these centers under extreme pressure. Starting in March, these centers ceased to receive medications, and by August 15, they were officially declared lacking essential cancer medicines such as chemotherapy, hormonal therapy, and targeted therapy. Furthermore, Sudanese oncologists and other supporting staff have not received any salary since the start of the military conflict. Nevertheless, they continue to provide services and serve as real-life examples of altruism. The scarcity of isotopes precipitated the collapse of nuclear services by early April 2023.

Radiation therapy was not widely available in Sudan before the war, and is even less available now. Originally equipped with two cobalt RT machines at the conflict’s onset, one ceased functioning in June, followed by the other in August. Consequently, as of August 15, the NCI was forced to discontinue RT services. Currently, the only center that offers RT to cancer patients is Eldaman Oncology and Radiotherapy Center in Merowe, Northern State. This center has one functioning linear accelerator radiation machine that belongs to the private sector. The expenses associated with RT, transportation and accommodation can’t be afforded by many cancer patients, forcing them to confront their impending death without proper care.

In Sudan, most cancer patients suffered from advanced or metastatic diseases. Among these individuals, uncontrolled pain was frequently mentioned as a prominent issue [5]. The supply chains and availability of opioid analgesics were disrupted due to the war, leading to a complete absence of these essential medications. Tragically, this has left cancer patients to endure excruciating pain without recourse, including the unavailability of opioid medications.

The limited access to oncology services during the current war endangers the lives of more than forty thousand Sudanese cancer patients [6]. The procurement and transportation of cancer medications are both intricate and costly endeavours. Given the burden of concurrent health issues and the destabilization of the Sudanese government, the provision of essential cancer medications presents an immense challenge. A collaborative effort involving various international organizations such as WHO and IAEA is imperative to secure basic cancer medications and facilitate the repair of radiation machines. The global health community bears a responsibility to intervene and ensure that Sudan’s cancer patients are afforded their fundamental right to access essential medications or, at the very least, the right to die with dignity and peace.

## Author contributions

All authors contributed equally to this Editorial.

## Conflicts of interest

We declare no competing interests.

## Funding

We did not receive any funds for this work
